# Effects of Thermal Stress on Growth and Reproduction of *Procambarus clarkii* and Aquaculture Best Practices

**DOI:** 10.3390/ani16030495

**Published:** 2026-02-05

**Authors:** Peipei Wang, Jackson Samwel Bakari, Yanqiu Han, Honghui Hu, Zhilong Liu, Yewei Zhang, Zigui Chen, Chungui Huang, Miaomiao Wang, Huangen Chen, Xiaojun Jing, Shengyan Su

**Affiliations:** 1 Wuxi Fisheries College, Nanjing Agricultural University, Wuxi 214081, China; bonniee.wang@outlook.com (P.W.); jbakari@ceelstz.or.tz (J.S.B.);; 2 Guangxi Introduction and Breeding Center of Aquaculture, Nanning 530001, China; 3 Key Laboratory of Integrated Rice-Fish Farming Ecology, Ministry of Agriculture and Rural Affairs, Freshwater Fisheries Research Center, Chinese Academy of Fishery Sciences, Wuxi 214081, China; 4 Jiangsu Fisheries Extension and Technology Center, Nanjing 210017, China; 5 Panjin Raoyang Agricultural Technology Development Co., Ltd., Panjin 124107, China

**Keywords:** red swamp crayfish, thermal stress, TRP channels, thermoregulation, sustainable aquaculture, rice–crayfish co-culture

## Abstract

Temperature is a critical abiotic factor regulating the physiology, growth, and reproduction of ectothermic aquatic animals. In China, the rapid expansion of the red swamp crayfish *Procambarus clarkii*) industry faces significant challenges due to seasonal temperature fluctuations (optimal growth at 20–25 °C and reproduction favored at 18–22 °C). This review focuses specifically on TRP channels, particularly TRPA1 as a key thermosensor in crayfish, and on downstream signaling pathways involving heat shock proteins (HSPs) and antioxidant defenses. We further link these biological mechanisms to aquaculture applications by evaluating best management practices for mitigating thermal stress, including integrated rice–crayfish co-culture, recirculating aquaculture systems (RAS), molecular marker-assisted breeding for thermal tolerance, and nutritional modulation (e.g., probiotics and immunostimulants). By maintaining thermal stability within the optimal range and directly enhancing physiological resilience through genetic and nutritional interventions, these practices provide a foundation for more sustainable and climate-resilient crayfish aquaculture.

## Simple Summary

Temperature strongly influences the growth, reproduction, and survival of aquatic animals, especially ectothermic species such as the red swamp crayfish (*Procambarus clarkii*), which is widely farmed. Seasonal temperature fluctuations and extreme thermal events caused by climate change increasingly threaten crayfish production and sustainability. Understanding how crayfish sense and respond to temperature changes is therefore essential for improving aquaculture management. This review summarizes current knowledge on the effects of temperature on growth performance, reproductive development, and the physiological health of *Procambarus clarkii*. Particular attention is given to transient receptor potential (TRP) channels, including transient receptor potential ankyrin (TRPA), transient receptor potential vanilloid (TRPV), and transient receptor potential melastatin (TRPM), which act as key temperature sensors and initiate downstream cellular responses. The activation of these channels can regulate calcium signaling, stress-related pathways, antioxidant defenses, and immune responses, helping crayfish adapt to thermal stress. In addition to biological mechanisms, this review discusses practical strategies to reduce temperature-related risks in crayfish farming. These strategies include rice–crayfish co-culture systems, improved water temperature management, selective breeding for thermal tolerance, and nutritional interventions. By linking molecular mechanisms with aquaculture practices, this review provides valuable insights for developing more resilient and sustainable crayfish farming systems under changing environmental conditions.

## 1. Introduction

Climate change has profound effects on temperature variability in aquatic ecosystems, with significant implications for crayfish production. Lower temperatures associated with cold weather affect both freshwater and marine environments [1], disrupting the breeding cycles and growth performance of temperature-sensitive species such as *Procambarus clarkii*. In China, *P. clarkii* production has expanded rapidly over the past decade, with annual output exceeding 2.8 million tonnes, accounting for the majority of global crayfish production and making China the world’s largest producer of this species [2,3]. This expansion has been largely driven by the widespread adoption of integrated rice–crayfish co-culture systems, which now dominate current farming practices and encompass extensive pond culture, rice–crayfish rotation, and regionally high-density monoculture under semi-intensive management. As a consequence, crayfish production is highly dependent on seasonal and interannual water temperature fluctuations. Deviations from optimal thermal conditions impose physiological stress that reduces productivity and leads to economic losses, whereas suitable temperatures support higher yields [4]. Moreover, elevated and extreme water temperatures exacerbate mortality and reduce survival, while also facilitating the spread of parasites and diseases that negatively affect crayfish populations [5].

Water temperature strongly shapes the growth patterns and spatial distribution of crayfish, with seasonal fluctuations evident in both winter and summer. Winter temperature, in particular, accounts for a large proportion of the variability observed in adult crayfish populations in lakes, although the underlying mechanisms remain unclear [6]. Across ponds, lakes, and rivers, water temperature and population density jointly regulate feeding activity, growth, and survival in crayfish irrespective of species [7]. For example, adult female noble crayfish exhibit high survival rates (91–98%) at temperatures of 8–10 °C from November to April, while juveniles maintained indoors at low densities and elevated temperatures (>17 °C) achieve survival rates of up to 94.5% over the same period [7].

Aquatic poikilotherms are highly vulnerable to rapid temperature fluctuations. In aquaculture, extreme cold or heat directly impairs crayfish immune function and antioxidant capacity Aquatic poikilotherms are highly vulnerable to rapid temperature fluctuations. In aquaculture, extreme cold or heat directly impairs crayfish immune function and antioxidant capacity [8]. Changes in environmental temperature influence the metabolism of aquatic animals, dissolved oxygen levels, and other factors, increasing the susceptibility of crustaceans such as *Procambarus clarkii*, shrimp, and *Scylla serrata* to infections. These fluctuations can also disrupt normal physiological functions and weaken immune defenses at the cellular and tissue levels [9]. As ectotherms, crayfish can withstand temperature variations during daily and seasonal cycles, but rapid or extreme temperature shifts beyond their normal range cause stress [10,11]. Such stress may result in slowed growth, reproductive diapause, impaired metabolism, pathological reactions, or death [12]. A significant challenge for aquaculture in northern China is managing high summer and low winter temperatures; summer heat often leads to increased disease outbreaks and mortality in aquatic animals [13].

*Procambarus clarkii* belongs to the Arthropoda, Crustacea, Decapoda, which also includes shrimp, crabs, and lobsters [3]. These freshwater lobsters are widely distributed in lakes, rivers, streams, and other freshwater habitats worldwide [14]. It is native to northern Mexico and the southern United States. However, it has spread worldwide due to its strong ability to invade new areas, its economic value, and accidental or intentional introductions [15,16]. Introduced to China from Japan in the 1930s, it has seen evolving farming models [17] in provinces with temperate and subtropical climates. By eating other invertebrates, macrophytes, algae, and detritus, crayfish contribute significantly to the freshwater food chain. Thomas et al. demonstrated that crayfish also exhibit cannibalistic tendencies and have a selective diet, focusing on particular macrophytes and invertebrates [18,19]. When crayfish are in great abundance, they both directly consume other species and, as a consumable, contribute significantly to the food chain and species diversity [20]. They hold a prominent place in the upper trophic levels of the food chain because they provide much-needed energy for many predatory animals. Crayfish represent, therefore, a naturally occurring food source that is economically significant [21].

Aquatic animals, including crayfish, are highly sensitive to changes in water temperature. Exposure to low temperatures induces physiological stress, resulting in reduced growth rates, diminished antioxidant capacity, and compromised immune function. Conversely, antioxidant enzyme activity is modulated by both the intensity and duration of high-temperature stress [22]. Prolonged exposure to elevated temperatures weakens immune defenses and increases susceptibility to bacterial, viral, and other pathogenic infections, thereby elevating disease incidence and mortality [23]. Crayfish generally perform best within a water temperature range of 12–30 °C, with optimal growth observed between 21 and 28 °C, whereas temperatures exceeding 33 °C markedly increase mortality [24]. Similar temperature-driven reductions in growth and survival have been reported in other crustaceans, including *Eriocheir sinensis*, *Macrobrachium nipponense*, and *Penaeus merguiensis*, particularly under fluctuating thermal conditions [25].

Under accelerating climate change, the development of climate-resilient aquaculture systems has become a priority for sustaining crayfish production. Such systems emphasize thermal adaptability, stress tolerance, and flexible management strategies to buffer the impacts of temperature extremes and variability, thereby enhancing both productivity and long-term sustainability.

This review integrates current knowledge of thermal stress on Procambarus clarkii’s biological functions and outlines best practices for resilient aquaculture. We review how temperature affects *P. clarkii* physiology (Section 1, Section 2 and Section 3), summarize the roles of thermosensory TRP channels (Section 4), and discuss downstream physiological responses (Section 5). Finally, we connect these mechanisms to aquaculture strategies such as co-culture systems, Recirculating Aquaculture Systems (RAS), selective breeding, and nutrition (Section 6). This structure was explicitly added to guide readers. By combining molecular physiology with applied aquaculture science, the review offers a framework for sustainable and climate-resilient crayfish production systems.

## 2. Mechanism of Thermoregulation in Crayfish

Metabolic rate and function are strongly temperature-dependent. Elevated temperatures can destabilize proteins and induce cellular damage, whereas temperatures below critical thresholds impair nervous system function and cellular metabolism [26]. To cope with thermal variability, animals have evolved diverse thermoregulatory strategies. In endotherms, these include autonomic mechanisms such as shivering and brown adipose tissue–mediated heat production, whereas ectothermic aquatic animals primarily rely on behavioral thermoregulation to maintain body temperature within a favorable range [27,28,29,30,31].

Crustaceans such as *Procambarus clarkii* lack endogenous heat production and therefore depend largely on behavioral strategies to regulate body temperature. These behaviors include navigating thermal gradients, selecting favorable microhabitats, and modifying activity patterns to avoid thermal extremes [32,33,34]. In natural habitats, crayfish frequently occupy shallow pools, streams, and littoral zones where water temperature can fluctuate substantially due to solar radiation and seasonal changes [35,36]. Consequently, the ability to locate and remain within suitable thermal environments is critical for maintaining metabolic efficiency, growth, and reproductive performance.

Behavioral thermoregulation through habitat selection is well documented in aquatic ectotherms. Although studies in fish and model organisms such as *Caenorhabditis elegans* and *Drosophila* have revealed general principles of thermal navigation—such as biased movement toward preferred temperatures and modulation of run length and turning frequency—these systems primarily serve as conceptual frameworks [26,37,38]. In crayfish, thermoregulatory behavior is shaped by species-specific ecological contexts and physiological constraints. *P. clarkii* actively searches for thermally favorable areas, seeking warmer shallow waters or burrowing into sediment to avoid extreme cold during winter, while moving to deeper or shaded habitats during summer to prevent overheating [39,40,41].

Burrowing represents a particularly important thermoregulatory strategy in crayfish. By constructing shelters within the sediment, crayfish can buffer against rapid or extreme temperature changes, maintaining a more stable microenvironment when surface conditions become unfavorable [40]. Such behaviors directly link thermal conditions to survival and seasonal activity patterns in crayfish populations.

Importantly, behavioral thermoregulation depends on the ability to detect environmental temperature changes and translate sensory input into adaptive actions. This process begins with peripheral thermal sensing, followed by neural integration and motor output. In crayfish, specialized sensory cells located in the antennae and other peripheral structures detect temperature fluctuations and relay this information to the nervous system [42]. These sensory signals initiate behavioral responses such as habitat selection, burrowing, or movement along thermal gradients. Additional environmental cues, including light and water flow, may further modulate these behaviors and refine thermal decision-making [42,43].

In crayfish, while the central integration of thermal signals is less complex than in vertebrates, the fundamental principle holds: peripheral thermosensation via specialized receptors initiates neural signals that are processed to drive adaptive behaviors such as habitat selection and burrowing [1,4,42]. Understanding how crayfish integrate thermal cues at the sensory and neural levels provides an essential foundation for exploring the molecular mechanisms of thermosensation, including the role of temperature-sensitive ion channels, which are discussed in the following section.

## 3. Effects of Temperature on Growth and Reproduction

### 3.1. Metabolism and Growth Patterns

**Figure 1:**
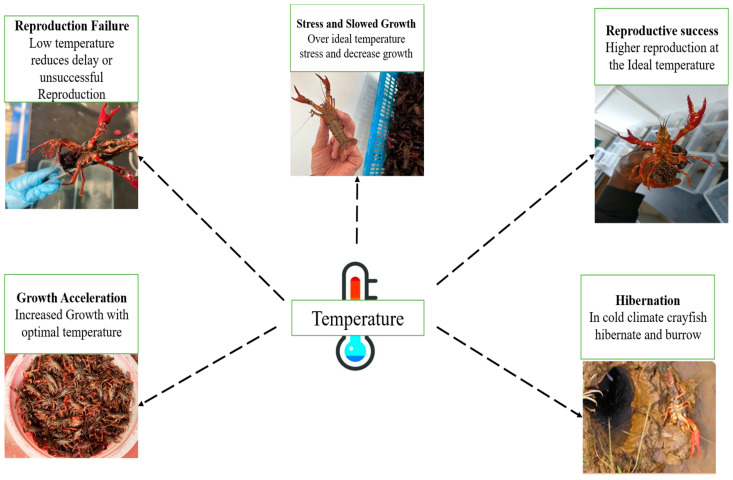
Schematic overview of temperature effects on growth, reproduction, and survival of *Procambarus clarkii*. Illustration in the lower right corner of the inset image reproduced from a public news website. Source: 12371 News [62], Cropped/adapted from the original.

Water temperature serves as the primary abiotic factor regulating the physiology of crayfish. As ectotherms, their metabolic rates, and consequently their growth and survival, are directly governed by the thermal environment [44,45]. While metabolic rates generally increase within an optimal thermal window, deviations from this range can be detrimental. The thermal niche varies significantly among species, reflecting adaptations to their native climates (Table 1). For instance, the temperate European crayfish (*Astacus astacus*) exhibits a lower optimal range (18–22 °C) and maximum tolerance (~25 °C) compared to the more eurythermal *Procambarus clarkii*, which thrives between 20 °C and 25 °C and tolerates up to ~32 °C [3,7,46]. For *P. clarkii*, temperatures exceeding 30 °C induce stress, reducing feeding efficiency and growth, while extreme heat (>35 °C) triggers burrowing behavior for survival (Figure 1). Conversely, temperatures below 15 °C lead to metabolic depression and stunted growth [47]. When temperatures fall below 5–8 °C, *P. clarkii* enters a state of hibernation and dormancy [44].

### 3.2. Reproductive Biology and Thermal Thresholds

Temperature critically influences reproductive success, dictating the timing of gametogenesis, mating, and spawning [55]. *P. clarkii* is a gonochoristic species with direct development, lacking a free-living larval stage, which simplifies aquaculture [56,57]. Sexual maturity is reached at approximately 18 g. Anatomically, the male system connects testes to the fifth pair of walking legs [58,59], while female oviducts open at the third pair [60]. Although females can spawn 3–5 times annually with high fecundity (100–1000 eggs) at temperatures above 22 °C [59], the optimal window for successful reproduction—balancing spawning frequency with offspring survival—is narrower [61]. Research indicates that while warmer conditions accelerate egg development [60], successful reproduction is typically confined to 18–22 °C, with activity ceasing below the 10 °C threshold [44].

### 3.3. Stage-Specific Thermal Requirements

The thermal requirements of *P. clarkii* shift dynamically across its life cycle. Early life stages have distinct thermal optima compared to adults [55]. Hatchlings require higher temperatures, with optimal growth occurring around 30 °C; notably, 15 °C is fatal at this stage [48,52,63]. As they develop into juveniles, the optimal growth range shifts slightly lower to 24–28 °C. Survival and growth rates for juveniles drop significantly below 20 °C or above 34 °C [55].Generally, development time from egg extrusion to the juvenile stage decreases as temperature increases, provided the temperature remains within the species’ physiological tolerance limits [63].

## 4. Adaptive Mechanisms and the Role of TRPs in Temperature Sensing in Crayfish

### 4.1. Adaptive Mechanisms of TRPs in Crayfish

The ability to sense ambient temperature is crucial in distinguishing whether an environment is safe to inhabit or harmful to avoid. For example, TREK-1, DEG/EnaC-family, P2X3, and the TRP family proteins are ion channels that mammals use to sense temperature signals [13,64]. Many members of a TRP ion channel superfamily have been discovered to be activated under temperature variations [65]. TRP channels, a large family of ion channels, play an important role in sensing environmental stimuli, including temperature [66]. These channels are present in a wide variety of organisms, such as crayfish [67]. They are essential for thermosensation, the ability of organisms to sense temperature changes. There are several subfamilies of TRP channels, and temperatures across different ranges can potentiate or inhibit the activation of their members.

#### 4.1.1. TRPA (Ankyrin) Family

TRPA1 is a temperature-responsive, nonselective cation channel belonging to the TRP family [68]. Different species exhibit varying levels of TRPA1 expression in their tissues [69]. This protein has been observed in various physiological contexts, including the heart, nervous system [70], respiratory tract [71], pancreas, reproductive tissues, inner ear [72], pancreatic islets [73], muscle [74], T cells [75], vascular endothelial cells [76], and diverse fibroblast types [77]. Consequently, TRPA1 plays multiple roles in these tissues or cells, including acting as a temperature and cold sensor [78] and participating in immune responses [78].

In crayfish, TRPA1-1 expression was detected in hemocytes, hepatopancreas [75], nerve, and muscle, with the highest levels in the hepatopancreas and nerve [79]. This provides clear evidence that PcTRPA1-1 is important for the hepatopancreas and nervous system (Table 2). To understand how silencing PcTRPA1-1 affects crayfish responses to high temperatures, the expression in the nervous system suggests it may function as a temperature sensor [80]. The main components of the crayfish immune system are hemocytes and the hepatopancreas [68]. Prober et al. found that the function of TRPA1 may differ between invertebrates and mammals [81]. It has been shown that TRPA1 acts as a thermal and cold sensor in various species. Exposure to 32 °C, but not 10 °C, induces PcTRPA1-1 expression, suggesting that PcTRPA1-1 may serve as a heat sensor but not a cold sensor. In humans and other mammals, TRPA1 functions as a temperature and cold sensor [82,83,84].

#### 4.1.2. TRPV

TRPV channels are ion channel proteins closely associated with temperature sensing, heat-induced nociception, and chemical irritants such as capsaicin [81,90,91]. These channels play essential roles in transmitting thermal and pain-related signals and are also involved in diverse physiological processes and disease development [82,90,91]. Members of the TRPV family are activated by warm to hot temperatures, with different subtypes responding to distinct thermal thresholds. The expression and activity of TRPV channels are modulated by multiple factors, including temperature, pharmacological agents, and intracellular signaling pathways [84,92]. Consequently, functional analyses of TRPV genes have been widely used to elucidate their regulatory mechanisms and signaling pathways in temperature-related physiological processes [83,93].

In crustaceans such as *Procambarus clarkii*, this gene is not well documented in many research reports due to the complexity of crustacean genomes and technological limitations, while most studies on TRPV genes have focused on vertebrates such as Mus musculus and Homo sapiens [94]. Therefore, their role in crayfish thermosensation is currently inferred from vertebrate physiology and the presence of TRPV homologs in crustacean genomes [80,92].

#### 4.1.3. TRPM8

TRPM8, a member of the transient receptor potential (TRP) ion channel family, functions as a molecular detector of cool and cold stimuli and plays a central role in the sensory transduction of temperature in many vertebrates and other taxa [64,95]. TRPM8 is activated by cooling within a broadly reported temperature window (approximately 8–28 °C) and can also be opened pharmacologically by cooling agents such as menthol, consistent with its role as a cold sensor. In mammals, TRPM8 is primarily expressed in sensory neurons and contributes to encoding cold sensations; additionally, in mammalian prostate epithelial cells TRPM8 expression is androgen-regulated and has been implicated in Ca^2+^ homeostasis [96,97].

Evidence for TRPM8’s role in crustaceans remains indirect. Comparative genomic and transcriptomic surveys detect TRPM8-like sequences in some crustacean genomes and suggest expression in sensory tissues [80,98], but direct functional characterization in crayfish (e.g., electrophysiology, calcium imaging, or loss-of-function studies) is currently lacking. Mechanistically, TRPM8 activation produces Ca^2+^ influx, which in vertebrate sensory neurons modulates excitability and can trigger release of neuropeptides such as CGRP and substance P that participate in nociception and inflammatory signaling [99]. Extrapolating these cellular mechanisms to crayfish is plausible but should be presented as a hypothesis rather than established fact [97,98].

Several studies in vertebrates indicate that TRPM8-expressing neuronal subpopulations contribute to sensory discrimination between innocuous and noxious cold, depending on activation thresholds and stimulus–response properties [100,101]. For ectothermic crustaceans such as crayfish, however, thermoregulatory responses are expected to be primarily behavioral (e.g., microhabitat selection, locomotor changes) in contrast to the autonomic responses described for endotherms (e.g., shivering, sympathetically mediated cardiovascular changes) [102,103]. Therefore, while TRPM8-mediated signaling could plausibly support cold-avoidance or other behavioral adjustments in crayfish, direct physiological and behavioral studies are required to confirm this.

### 4.2. Role of TRPs in Detecting Temperature Changes

#### 4.2.1. Immune Response

TRPA1 functions as a temperature sensor, detecting changes in temperature and signaling the crayfish immune system to adapt to higher temperatures. Through both direct and indirect mechanisms, TRPA1 supports crayfish immunity. It directly influences animals’ immune responses. Previous research has shown that TRPA1 is crucial for inflammatory reactions triggered by environmental and pharmacological factors [104] and that it contributes to the development and worsening of asthma [71]. Wang et al. [105] also indicate that TRPA1 plays a role in atherosclerosis development. Additionally, De Logu et al. suggest that Schwann cell TRPA1 is necessary for neuroinflammation [106].

*Procambarus clarkii* immune responses are directly impacted by TRPA1 silencing, as it can reduce PO activity, a key immune metric for crayfish [107]. PcTRPA1-1 silencing also influences the expression of *Plasmodium chabaudi* Heat Shock Protein (PcHSP70 and PcHSP90), which are associated with heat stress and crustacean immune responses [108]. The evidence that PcTRPA1 silencing can directly affect crayfish immune responses is further supported by the reduced expression of PcHSP70 and PcHSP90 at higher temperatures. The high mortality rate of crayfish with silenced TRPA1 at elevated temperatures results from decreased expression of PcHSP70 and PcHSP90.

#### 4.2.2. Homeostasis

Thermoception and thermoregulation are mainly facilitated by TRP channels, which play a crucial role in maintaining homeostasis during changes in temperature. These sensors, found throughout various tissues, detect environmental temperature changes and enable appropriate physiological responses to preserve internal balance. Since crayfish cannot regulate their body temperature through metabolism like warm-blooded animals do, their ability to sense temperature fluctuations in their surroundings is vital [109]. To maintain internal stability, it is helpful to use TRP channels to detect external temperature changes and adapt behavior or physiology accordingly. For example, if water temperature shifts, crayfish may modify their behavior by activating specific TRP channels to continue everyday life activities.

There are various subtypes in the TRP channel family, including TRPV1, TRPV2, TRPA1, TRPM8, and others. Each subtype has its own temperature sensitivity profile. For example, TRPV1 is activated by noxious heat (>43 °C), menthol, or cool temperatures (<25 °C) [110,111]. This diversity enables organisms to detect a range of thermal stimuli vital to survival. Activation of these channels initiates intracellular signaling cascades that modulate neuronal excitability and drive thermoregulatory behavioral responses [111,112].

In the physiological context of the skin, TRP channels are key players in activating HSPs during heat stress. HSPs protect cells by stimulating repair mechanisms and preventing protein denaturation. Activation of TRP channels in the skin helps maintain thermal homeostasis by enabling the sensation of temperature changes and triggering protective responses that increase cellular resilience to environmental stress [113,114]. Additionally, TRP channels regulate ion homeostasis, which may help sustain cellular activity across different temperature ranges. They also facilitate the passage of cations like calcium and magnesium, both of which are essential for processes such as neurotransmitter release and muscle contraction [109,115]. Their importance in health and disease is underscored by the fact that dysregulation of TRP channel function can lead to pathological conditions [116].

#### 4.2.3. Sensory and Behavior Response

Crayfish rely on TRP channels to detect temperature changes, which actively modulate their behavior. For instance, research has indicated that crayfish alter their heart rate under thermal stress [117,118], but it also indicates that heart rate can affect movement in response to temperature changes. This physiological response is vital for maintaining metabolic balance and ensuring proper functionality across various temperature environments in behavior, whereas activation of TRP channels is the crucial mediator of crayfish’s unique responses to temperature changes. For example, at high temperatures, crayfish often display avoidance behavior, such as seeking cooler sites or reducing activity levels to prevent hyperthermia [119]. These behavioral adaptations are vital for avoiding stress and maintaining homeostasis in their aquatic habitats. Crayfish have been shown to alter their social interactions and foraging strategies in response to temperature cues as well, indicating a complex integration of sensory input to guide behaviors [120].

**Figure 2:**
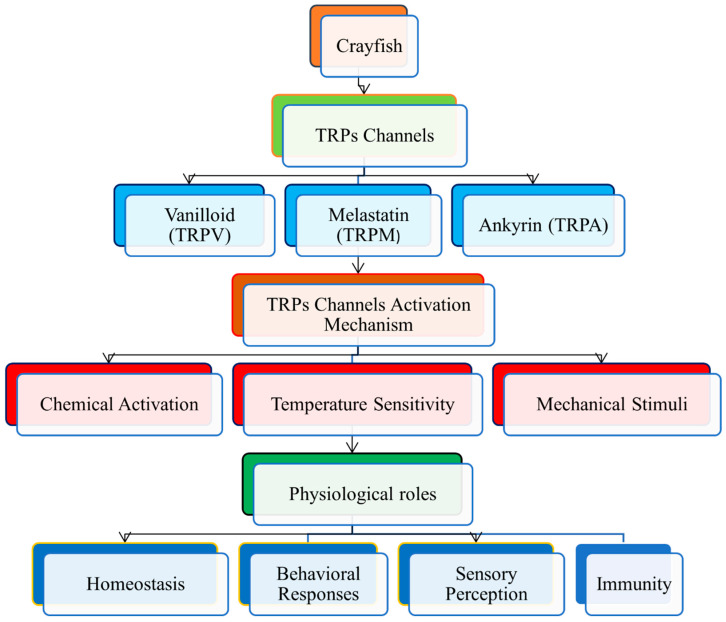
Types of TRP channels involved in temperature sensing and physiological regulation in crayfish.

In addition, social behaviors in crayfish are shaped through interactions between TRP channels and environmental stimuli. Research on the influence of temperature and other environmental factors on crayfish behavior in the context of competition and mating has shown that these conditions can be manipulated, leading to altered interactions between crayfish [121]. They rely on their ability to sense the right conditions and adjust behavior, especially in variable environments like ours, where temperatures can change rapidly. Overall, TRP channels are essential for crayfish thermos sensation, the ability to detect temperature deviations, followed by adequate behavioral and physiological responses (Figure 2). Such reactions are critical for maintaining homeostasis and optimizing survival in their natural environments.

## 5. Molecular Mechanisms of Crayfish Under Thermal Stress

The response to temperature extremes through gene–protein interactions creates a complex variety of thermal stress facing crustaceans. The activity of specialized ion channels (TRP) is a prominent feature of this, as evidenced by temperature sensitivity and chemical stimulation. Acute temperature changes prompt this animal to retreat from high temperatures to icy environments; however, not all chemical stimuli elicit the same degree of withdrawal. Consequently, *Procambarus clarkii*’s mechanical (heat) senses exhibit brief, high, transient bursts likely attributed to these ion channels as a direct response to extreme thermal exposure [122].

The TRP Channel (TRPA1) becomes more likely to open as temperature increases, and the higher likelihood of it opening due to temperature is caused by an increase in the amount of thermal energy available to change the channel’s gating “equilibrium”, which is known as “enthalpy-dependent channel gating.” The average openings of TRPA1 at high temperatures result from the combined functions of the ankyrin repeat domain, the S1–S4 voltage-sensor-like structure, and the S5–S6 pore [123]. Additionally, TRPA1 is thermosensitive, which varies by class (i.e., insect versus mammal), but many insects use TRPA1 to detect temperature and trigger flight or avoidance responses.

TRPA1 channels conduct Na^+^ and Ca^2+^ ions when open, leading to depolarization of antennal sensory neuron membranes and an increase in intracellular Ca^2+^ levels. More vesicle fusion events and enhanced synaptic transmission to second-order interneurons in the crayfish CNS escape circuit follow. TRPA1 is likely involved in regulating the downstream signaling process described above in crayfish, and there is substantial evidence supporting TRPA1’s role in thermosensory behavior, thermal fitness, and related gene/function interactions in both insects and mammals [69].

A TRPA1 paralog (PcTRPA1-1) in *Procambarus clarkii* is upregulated by high temperature and is necessary for survival following bacterial exposure at 32 °C. Silencing of PcTRPA1-1 impairs high-temperature defense and supports the idea that PcTRPA1-1 functions as a temperature sensor, likely involved in stress and immune pathways [69]. Thermal detectors are present in the antennae and antennules, the most direct method of sensing the environment, aligning with the distribution of chemosensory and mechanosensory TRPA1s reported in the appendages of decapods, indicating that thermal nociception likely originates from the periphery [122].

The evidence supporting the role of specific TRP channels in crayfish thermosensation varies in strength and methodology. While TRPA1 has been directly investigated in *Procambarus clarkii*, evidence for other TRP subfamilies (e.g., TRPV, TRPM) is largely inferential, derived from comparative genomics or extrapolated from vertebrate models. Table 3 synthesizes the available experimental and comparative evidence, highlighting the established role of TRPA1 as a heat sensor and nociceptor in crayfish, while also noting the candidate status of other TRP channels based on studies in related crustaceans and distant taxa.

TRP thermal gating is a flexible system; it allows organisms to alter their TRPA1’s likelihood of opening (to heat) through various means (PIP_2_ & other phosphoinositides; redox/electrophilic adducts; and kinase pathways [PKA/PKC]) across different species among invertebrates. In ectothermic species, this plasticity enables fine-tuning of TRPA1’s thermal sensitivity, providing the organism with the ability to adjust its thermal response to stressors (infections), as well as to dietary shifts and/or inflammation. The number of crayfish-specific modulation studies is limited; however, the conserved mechanisms identified in these studies offer an important model for understanding the plasticity of the thermal sensitivity threshold of *Procambarus clarkii* [80].

The phenotype of PcTRPA1-1 silencing during infection at 32 °C demonstrates that Ca^2+^-dependent signaling from TRPA1 to unfolded protein and oxidative stress pathways, as well as the expression of immune effector molecules, is analogous to the molluscan TRPA1-like functions, indicating an ancient evolutionary origin of the thermal stress response axis [69]. Central to this response are HSPs, which are vital for protecting cells against thermal stress. These proteins serve as molecular chaperones, assisting in proper protein folding and preventing aggregation caused by high temperatures [123,130]. For example, the increased expression of HSP70 and HSP20 in response to heat stress highlights their role in stabilizing proteins under stress [124,125].

The impact of thermal stress on *Procambarus clarkii* is evident in changes in transcriptional and proteomic profiles related to stress response, as shown by recent transcriptomic investigations that identify considerable changes in gene expression linked to stress responses, especially in muscle tissues and the hepatopancreas [113,116]. The influence of cold thermal stress on metabolic pathways was also highlighted in these studies, where many genes with increased expression are linked to lipid metabolism, such as sphingolipid 4-desaturase. This indicates that crayfish exposed to temperature fluctuations undergo metabolic changes [116]. Additionally, changes in metabolic pathways have been shown to lead to higher energy demands and alterations in membrane fluidity, which can impact cellular homeostasis [115].

**Figure 3:**
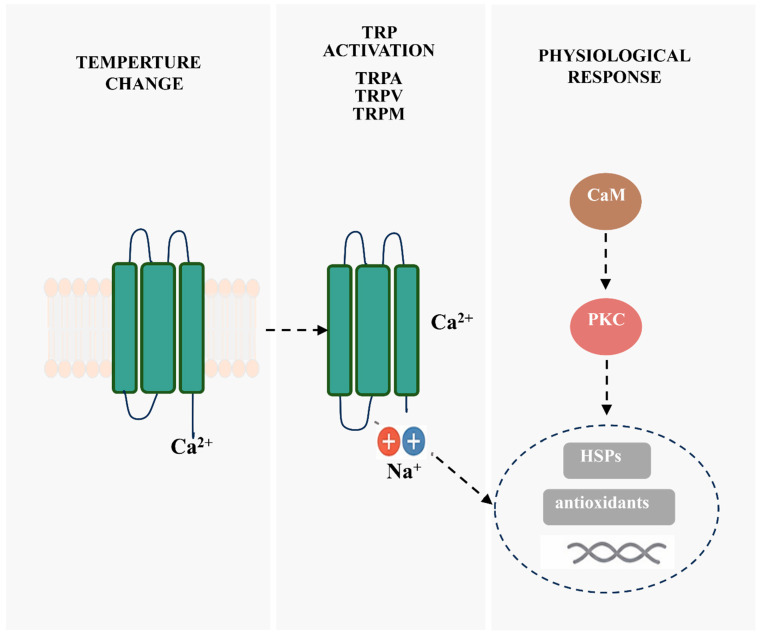
Proposed molecular mechanisms underlying temperature-stress sensing and downstream physiological responses in crayfish. Thermal stress activates thermosensitive TRP channels (mainly TRPA, TRPV, and TRPM) on the membranes of sensory and epithelial cells, leading to Ca^2+^ and Na^+^ influx. Elevated intracellular Ca^2+^ activates CaM and PKC, triggering downstream signaling pathways that regulate gene transcription. This results in the induction of HSPs and antioxidant enzymes, enhancing cellular protection and resistance to oxidative stress.

The integrated molecular response to thermal stress in crayfish can be summarized as a signaling cascade initiated by thermosensitive TRP channels. As detailed above, thermal stress activates TRP channels (notably TRPA1) on sensory and epithelial cell membranes, leading to Ca^2+^ and Na^+^ influx. This elevated intracellular Ca^2+^ activates downstream mediators such as calmodulin (CaM) and protein kinase C (PKC), which in turn regulate gene transcription. The transcriptional output includes the induction of HSPs and antioxidant enzymes, which collectively enhance cellular protection, stabilize proteins, and mitigate oxidative damage [69,123,124,128]. This proposed pathway is illustrated in Figure 3.

In addition to the general stress response to higher temperatures, organisms also use molecular signaling mechanisms to adapt to different temperature extremes. For instance, Heat Shock Transcription Factor (HSF1) is a key regulator of gene expression for HSPs produced during heat stress [127]. The expression of HSPs influences how cells respond to oxidative stress and helps with cellular repair and recovery after heat-induced damage [124,127]. Additionally, at extreme temperatures, oxidative stress becomes a major concern, as an increase in reactive oxygen species (ROS) can damage cells, which increases the need for effective antioxidant protection [128].

As observed in crayfish experiencing thermal stress, the cellular antioxidant response (AR) indicates an integrated stress response system that includes both HSPs and antioxidant enzyme activity working together to minimize oxidative damage and aid in repairing damaged cells [131]. To date, most research has focused on how various temperature-induced physiological responses are affected by single stress factors. Furthermore, it is increasingly recognized that thermal stress interacts with osmotic or oxidative stress, resulting in integrated cellular and molecular responses [128,132].

In summary, thermosensation in *Procambarus clarkii* involves a hierarchy of molecular players with varying levels of experimental support. TRPA1 emerges as a central, experimentally validated heat sensor linking environmental temperature to immune and stress pathways [69]. The potential roles of TRPV and TRPM8, while physiologically plausible based on comparative models [80,97], require direct functional confirmation in crayfish. The activation of these channels, particularly TRPA1, initiates a conserved downstream cascade involving calcium signaling, HSP induction, and antioxidant responses, which collectively determine the organism’s capacity to cope with thermal stress [122,123,124]. The molecular response to thermal stress is not a linear pathway but an integrated network. The following Table 4 synthesizes current knowledge, linking specific thermal stimuli to their sensory mechanisms, downstream molecular effectors, and resultant physiological outcomes that are relevant for aquaculture management.

## 6. Best Practices for Crayfish Farming Under Thermal Stress

### 6.1. Hybrid or Integrated System

Integrated aquaculture systems, such as rice–crayfish co-culture, represent a holistic strategy to mitigate environmental stressors, including thermal fluctuations, by leveraging ecological synergies. This system functions as a buffer against thermal stress at both the environmental and organismal levels [136,137,138].

Around the world, many challenges exist, such as limited water resources, temperature fluctuations, and land degradation, creating a need for solutions in agricultural development. International proposals have suggested various models, including soilless vegetable cultivation, rice–fish farming, and dry direct-seeded rice technology, to address these issues [3]. Integrated crayfish farming with other organisms, like fish and plants (Rice–crayfish culture), helps create a more balanced ecosystem where different temperature preferences can be utilized for more efficient production. Beyond rice–crayfish systems, polyculture of *Procambarus clarkii* with fish species such as common carp (*Cyprinus carpio*) or tilapia (*Oreochromis* spp.) is practiced. This can improve resource use efficiency and system stability, though specific research on thermal benefits in such polyculture with *Procambarus clarkii* is limited compared to rice–crayfish systems. Critically, the rice canopy provides shade, which reduces diurnal water temperature extremes and solar radiation in the water column [139]. This moderated thermal environment can prevent crayfish from reaching critical temperature thresholds that trigger the TRPA1-mediated heat-stress response and the subsequent energetically costly upregulation of HSPs and antioxidant enzymes [69,124]. By dampening the amplitude of thermal fluctuation, integrated systems can reduce the metabolic demand associated with repeated stress acclimation.

This farming approach allows us to gain greater economic benefits by producing both rice and crayfish, especially in diverse climate zones. Subtropical regions see significantly greater benefits compared to tropical and temperate areas. When combined with climate effects, subtropical zones could significantly boost economic gains through models like the “one season rice with two seasons crayfish” or even the “one season rice with three seasons crayfish” production systems. Crayfish production may rise from 1500 kg per hectare in a single season to 2250 kg per hectare in two seasons. Due to limited arable land and increasing demand in recent years, paddy fields continue to be developed to produce both grain and aquatic products [113].

Furthermore, crayfish also assist in pest control within rice–crayfish co-culture systems. This occurs when crayfish feed on pests, promoting healthy growth and survival of rice as well as improving water quality [140]. This is a symbiotic relationship where crayfish need rice to help control pests, and rice benefits from waste products produced by crayfish to enhance water quality. From a physiological perspective, improved water quality—particularly stable dissolved oxygen and lower ammonia—reduces secondary environmental stress that can compound thermal stress and weaken immune function [9]. A stable, low-stress environment helps maintain robust immune and antioxidant capacities, which are crucial defenses that can be compromised under temperature extremes [8,22]. Integrated production systems contribute to higher field biodiversity and enable more efficient cycling of energy and nutrients within the ecosystem [141]. This includes fostering a diverse aquatic microbiome, which can positively influence the crayfish gut microbiome. An optimal gut microbiome has been linked to enhanced host metabolism, immune competence, and stress resilience, including improved cold tolerance through modulated histamine pathways [123,134]. Other reports indicate that internal recycling in rice–aquatic animal co-culture systems can significantly boost farmers’ income while reducing reliance on external sources of fertilizer, feed, and pesticides for food production.

### 6.2. Use of Artificial Heating and Cooling Methods

Artificial temperature control is a direct intervention to maintain water conditions within the optimal thermal range for *Procambarus clarkii* (approximately 20–25 °C, see Table 1). This practice targets the fundamental ectothermic physiology of crayfish by preventing the cellular stress responses triggered at temperature extremes.

Cold temperatures reduce growth, survival, and reproduction in many crustaceans, including *Procambarus clarkii*. To maintain optimal water temperature for species growth, different methods were used to stabilize water temperature and support health and growth. From a molecular perspective, preventing cold stress avoids metabolic depression, shifts in lipid metabolism, and potential dysregulation of ion homeostasis that occur at low temperatures [10,116,125]. Maintaining warmth supports standard metabolic function and prevents the activation of putative cold-sensing pathways (e.g., those potentially involving TRPM8-like channels) that could lead to behavioral inactivity and suppressed feeding. These methods, such as using heaters, are used to raise water temperature in winter climates. In aquaculture, the use of submersible heaters, which are set directly in the tank to raise water temperature to the ideal level. They can be impractical for large installations, but water baths can bring an entire tank to the desired temperature. Under-tank heaters use similar logic but can heat the water much more uniformly than a heating pad, since there is not a large temperature gradient from top to bottom. Boilers and heat exchangers are used on a large scale to heat water externally and circulate it throughout the tank system.

Conversely, active cooling during hot weather prevents heat stress, a condition with well-defined molecular consequences. Excess heat can directly denature proteins and increase oxidative stress. By keeping temperatures below critical thresholds (e.g., <32 °C), artificial cooling helps avoid the strong activation of heat-sensitive TRPA1 channels [69,122]. This, in turn, prevents the excessive and energetically costly induction of HSP70 and HSP90 and the overproduction of ROS that demand a robust antioxidant response [108,124,128]. Mitigating this stress preserves cellular energy budgets for growth and reproduction instead of stress repair.

Unlike Heating materials during hot weather, it is also essential to prevent excessively high water temperatures, as this can cause the crayfish to become stressed and even die. Chilled water systems operate in much the same way as heating systems; the water can be cooled externally before being returned to the tank. Also, air conditioning (AC) units are used to cool the air above the water surface and lower the water’s temperature. More advanced systems might use automated controls to monitor and adjust water temperature based on sensor readings. These systems can use the detected water temperature to program the heating or cooling mechanism to turn on or off. The physiological benefit of such automated systems is the prevention of rapid thermal fluctuations, which are particularly damaging as they can repeatedly engage and disengage stress-response pathways, leading to cumulative metabolic wear and impaired immune competence [8,9].

The application of these heating and cooling methods always depends on the species being farmed, the size of the farm, and the local climate. Other critical factors to consider are water quality and oxygen levels, as they can affect crayfish health and the efficiency of any cooling or heating systems used. Notably, temperature and water quality have synergistic effects. For instance, warmer water holds less dissolved oxygen, and thermal stress increases metabolic oxygen demand. Therefore, effective temperature management must be integrated with aeration to ensure that the physiological benefits of thermal stability are not undermined by hypoxic stress, which itself can trigger similar pathways of metabolic disruption and immunosuppression [9].

### 6.3. Recirculating Aquaculture Systems (RAS)

RAS represents the pinnacle of environmental control in aquaculture, offering the most direct technological intervention to decouple crayfish production from external climatic variability. The core physiological advantage of RAS lies in its capacity to maintain precise, stable water temperature, thereby avoiding the thermal stress responses detailed in previous sections.

A RAS is a multidisciplinary engineering method that combines aquaculture science, mechanical engineering, hydrochemistry, hydromechanics, biology, and electrical engineering. Through a number of water treatment procedures, this contemporary intensive aquaculture cleans aquaculture water and encourages recycling [142]. RAS typically consists of a water treatment unit with filtration devices to remove particulate matter (feces, leftover feed), biological reactors to remove nitrogen, phosphorus, and other pollutants from the water, oxygenation equipment, and sterilization devices, as well as an aquaculture unit for breeding aquatic products. The drawbacks of traditional extensive aquaculture methods are becoming more widely known as the industry grows. These drawbacks include an over-reliance on land and natural space, which can lead to water pollution, disease outbreaks, harm to the coastal ecosystem, and other uncontrollable factors that are detrimental to the sustainable growth of aquaculture [143,144,145].

RAS has several advantages, including the ability to be grown in a controlled environment, the ability to control the growth rate and harvest cycle, the ability to recycle water through bioreactor filtration, which requires water reductions of 90% to 99% less than consumption when using the traditional method, and the ability to occupy less than 1% of the land. There are also no environmental restrictions on the scale of cultivation, and it is simpler to withstand external risks (natural disasters, pollution, and disease). To guarantee safety, contaminants like chemical sand and heavy metals will not be added to aquaculture water. Crucially, this controlled environment extends to thermal management. By integrating heaters, chillers, and insulation, RAS can maintain temperatures within the optimal range for *Procambarus clarkii* year-round [48]. This stability has profound molecular implications: it prevents the activation of thermosensitive TRP channels (e.g., TRPA1) that initiate stress cascades, minimizes the metabolic cost of repeatedly inducing HSPs for acclimation, and avoids the oxidative damage associated with both heat and cold stress [69,123,128]. Since these systems can be enclosed, temperature can be adjusted through the use of climate control technology [48]. Adding insulation to tanks and pipes helps maintain stable temperatures in both hot and cold environments. In order to more effectively utilize the scarce local water resources, carp farming in Japan used biological filters to create RAS in the 1950s [146]. RAS are used to cultivate fish, crustaceans, mollusks, and *Echinodermata*, both marine and freshwater.

Furthermore, the superior water quality maintained in RAS characterized by stable pH, low ammonia/nitrite, and high dissolved oxygen creates a low-background-stress environment. This is critical because poor water quality can act as a secondary stressor, synergistically exacerbating the physiological load of suboptimal temperature and compromising immune function [8,9]. By minimizing these compounding stressors, RAS allows crayfish to allocate energy towards growth and reproduction rather than continuous stress mitigation. Advantages of RAS include:

- Rigorous consideration of local climate, ambient air and water temperature conditions, incoming water treatment, and biosecurity in the RAS design can mitigate risks associated with disease, parasite, and climate-related factors. This integrated biosecurity and environmental control directly supports immune competence, as a stable thermal environment prevents the temperature-dependent immunosuppression that increases susceptibility to pathogens like Aeromonas hydrophila [69,107].
- Allow the production of a wide variety of species regardless of the required temperature, as long as the costs associated with controlling the temperature above ambient are low. This flexibility is rooted in the ability to manipulate the organism’s molecular thermostat, enabling cultivation outside a species’ native thermal range by artificially providing its optimal physiological conditions.

The versatility and effectiveness of RAS for controlled crustacean cultivation are demonstrated by its successful application across a diverse range of species, as summarized in Table 5. This includes commercially significant species such as the Pacific white shrimp (*Litopenaeus vannamei*) and the European lobster (*Homarus gammarus*), as well as other decapods cultured under varied conditions [147,148,149,150]. The table illustrates that RAS technology is not merely theoretical but is actively implemented in research and commercial settings worldwide to optimize growth and mitigate environmental stressors, including thermal fluctuations, for numerous crustacean taxa.

### 6.4. Seasonal Farming and Breeding

Strategic management of breeding cycles and the development of thermally resilient genetic lines are proactive approaches to align crayfish production with thermal biology. These strategies aim to either synchronize life stages with favorable natural temperatures or genetically enhance the underlying molecular pathways that confer thermal tolerance.

Crayfish can breed at specific temperatures (typically around 20 °C to 30 °C). When temperature conditions are unfavorable, it may be best to time breeding and growth cycles to coincide with natural seasonal variations in temperature. This practice, known as seasonal farming, works by ensuring that thermally sensitive life stages such as reproduction, egg incubation, and juvenile growth occur within their optimal thermal window (Table 1). This avoids exposing these vulnerable stages to temperatures that would induce stress, suppress immune function, or cause developmental abnormalities [44,60]. From a molecular perspective, it circumvents the need for sustained activation of stress-response pathways (e.g., HSP induction, antioxidant defense) during critical developmental periods, thereby preserving energy for growth and gonad development.

To our knowledge, no large-scale genetic selection programs targeting thermal tolerance have been reported for *Procambarus clarkii* [161]. Breeding strategies and techniques, such as family selection, genomic selection, and marker-assisted selection using heat shock protein (HSP) genes and other candidate loci, could be employed to develop heat- and cold-tolerant strains of *P. clarkii*. In contrast, related crayfish species within the genus Cherax have undergone selective breeding programs focused on growth performance [162].

These days, one of the most prominent breeding techniques in aquaculture is family selection breeding. Numerous aquaculture species, including *Litopenaeus vannamei*, *Fenneropenaeus chinensis*, *Salmo salar*, and *Macrobrachium rosenbergii*, have been used in many research studies [162]. In molluscs, heat-tolerant strains or families have been developed in Pacific abalone and scallops through family-based selection and genomic selection for heat survival traits [163,164]. For *Procambarus clarkii*, genetic selection programs are emerging. Initial studies have estimated heritability for growth traits, providing a foundation for selective breeding [165,166]. Furthermore, marker-assisted selection (MAS) targets specific genes, such as those encoding HSP70 and HSP90, which have been associated with high-temperature tolerance and present potential targets for breeding more resilient strains [155]. Genetic parameter estimates are essential in family selection and breeding because accurate estimates can yield useful information for establishing sensible breeding strategies, forecasting selection response, and determining the breeding values of consanguineous traits [165]. Through obtaining usable traits that can be progressed in another generation with temperature-tolerant species.

The second method to help crayfish cope with the stress of high temperatures is to breed high-temperature-tolerant varieties using the germplasm resources. The high-temperature-tolerant varieties are obtained through Molecular Marker-Assisted Selection (MAS) breeding. MAS is a breeding technique with good results compared to traditional breeding methods. Aquatic animals with outstanding economic traits like stress resistance, growth, and quality are commonly cultivated through MAS breeding, which offers the advantages of being quick, precise, and free from environmental constraints [155]. Importantly, MAS allows for the direct selection of favorable alleles in genes central to the thermal stress response. This includes genes encoding for more efficient or resilient including HSP70 and HSP90, robust antioxidant enzymes, and even variants of thermosensory channels like TRPA1 that might alter thermal sensitivity thresholds [108,124,155]. By stacking these favorable alleles, breeding programs can enhance the integrated stress response, making selected lineages more capable of maintaining homeostasis during temperature fluctuations.

The number of individuals possessing two genetically preferred haplotype combinations is lower in natural crayfish populations. In order to create homozygous lines for each of the preferred haplotypes of a single gene, we first select the preferred haplotypes of that gene separately. Next, we hybridize the preferred haplotypes to create individuals with two gene variants that are preferred [155]. To create a preferred haplotype combination of homozygous crayfish, the resultant individuals can also undergo additional hybridization. By connecting more genes that are resistant to high temperatures, this technique can maximize the crayfish’s capacity to withstand high temperatures. Furthermore, hybridization can be utilized to combine various desirable features to produce a wider range of crayfish varieties. This approach essentially engineers a more resilient molecular toolkit within the crayfish. The culmination of this strategy is a broodstock whose offspring possess a genetic predisposition for a more effective cellular response from initial thermosensation and signal transduction to protein protection and oxidative defense when faced with thermal challenge.

### 6.5. Nutritional and Other Strategies

Targeted nutrition serves as a frontline intervention to bolster crayfish physiology against thermal stress (Table 6). By modulating the gut microbiome, enhancing antioxidant defenses, and providing substrates for critical metabolic pathways, dietary strategies can directly support the molecular and cellular systems challenged by temperature extremes.

Optimizing nutritional factors is one of the main strategies to develop crayfish disease resilience under thermal stress. Probiotics (such as lactic acid bacteria) can improve antioxidant capacity by regulating histamine metabolism, and research papers show a positive correlation with cold resistance, providing a target for nutritional intervention under thermal stress [134]. Specifically, a well-modulated gut microbiota can enhance the host’s endogenous antioxidant capacity, providing a crucial line of defense against the elevated ROS produced during both heat and cold stress [128,134]. This microbial support can mitigate oxidative damage to lipids, proteins, and DNA, thereby preserving cellular integrity and function. Dietary supplements like probiotics are beneficial in growth, immune response, and stress tolerance in crayfish. Probiotics are frequently used as feed and water additives in aquaculture, particularly in crayfish culture, and the best benefits of probiotic application depend on choosing the right strain. Application of Probiotics in Aquaculture [135]: Probiotics investigated in crayfish culture are typically isolated from pond sediments, water, and the host’s intestine. It has been shown that the application of the right probiotics can improve the intestinal microbiota, growth performance, and immune function of crayfish [12,139]. This improved immune function is critical under thermal stress, as both high and low temperatures can suppress innate immunity and increase susceptibility to pathogens [8,23]. Probiotics can help maintain immune competence, potentially compensating for the temperature-dependent immunosuppression linked to stress-response pathways. Dietary supplementation with *Clostridium butyricum* is associated with marked changes in gut microbiota composition and enhanced innate immune responses, underscoring the potential role of probiotics in supporting health under stress conditions [169,170]. *Cyanobacterial filaments* can also modulate gut microbiota and stimulate the immune response [170] much like the known role of *Lactobacillus* species in gut microbiota modulation which means both of them can be potentially beneficial as dietary probiotics during temperature changes.

Together with probiotics, the dietary composition of feed is significant. Diet protein content and quality directly affect the growth and reproduction performance of crayfish. As such, research demonstrates that the types of lipids added to the diet can have large effects on growth and gonad maturation in crayfish, especially pre-adult females [171]. Dietary lipids influence membrane fluidity and composition. Maintaining optimal membrane fluidity is a key challenge during cold stress, and tailored lipid supplements can support this aspect of cellular homeostasis, reducing the metabolic cost of acclimation [116,167,171]. Additionally, some studies found that juvenile crayfish fed biofloc technology had better performance of their immune and antioxidant enzymes, suggesting that alternative feed strategies could help cope with adverse environmental conditions [171]. Biofloc not only provides a sustainable protein source but also delivers bioactive compounds and a diverse microbial consortium that can further enhance gut health and systemic antioxidant status, directly supporting the physiological systems taxed by thermal stress [123,172,173].

Crayfish metabolic processes are also affected by thermal stress. Crayfish are known to alter their amino acid metabolism upon exposure to cold stress, and this amino acid profile is useful in reflecting muscle quality and whole-body health [123,173], and diets high with amino acids that support metabolic functions are good to consume. Also, other aspects, such as the combination of amino acids, such as valine and isoleucine, in the diet, may alleviate the effects of temperature through modulation of metabolic pathways that are essential for energy generation [123,168,173]. This is particularly relevant because thermal stress alters energy demand. Providing key amino acids ensures the availability of substrates for the de novo synthesis of HSPs and other stress-related proteins, as well as for energy production via gluconeogenesis or the TCA cycle [123,173]. Nutritional support thus fuels the very metabolic adaptations required for thermal tolerance.

Furthermore, integration systems such as rice–crayfish co-culture, which are key aquaculture strategies, help to solve the issue of temperature and make the field more sustainable and resilient to thermal stress. This not only diversifies the food of crayfish but also promotes the health of the aquatic ecosystem, thus reducing the stress response in crayfish populations [125,167,168]. The co-culture system has been demonstrated to enhance nutrient cycling and enhance microbiome compositions in crayfish which are extremely advantageous during thermal stress [123,174]. Thus, the nutritional benefits of co-culture are twofold: it provides a diverse, natural diet that supports a robust gut microbiome, and it creates an environment that minimizes background stressors, allowing nutritional investments to be directed towards growth and resilience rather than basic stress mitigation.

## 7. Conclusions

The review emphasizes that temperature is a key factor affecting the growth, reproduction, and survival of *Procambarus clarkii*. The effects of temperature changes are likely to place more physiological and metabolic stress on organisms, stress that can reduce immunity, decrease reproduction, and ultimately impact aquaculture production. In countering temperature effects, integrated aquaculture approaches involving the rice–crayfish co-culture system, RAS, probiotic addition, and even the breeding of temperature-tolerant strains using molecular methods are potentially key strategies for improving crayfish tolerance to extreme temperatures while also supporting environmental sustainability and improving production efficiency. A holistic approach to food production that incorporates molecular, environmental, and nutritional approaches will be necessary to develop resilient and sustainable crayfish aquaculture in the face of global climate change and the associated challenges.

Moreover, future research should focus on addressing key knowledge gaps to improve the precision and effectiveness of management strategies. Important areas of investigation include: (1) fully mapping the peripheral thermal sensing network in crayfish by functionally characterizing non-TRPA1 thermosensors (e.g., TRPV and TRPM channels); (2) developing molecular markers linked to thermal tolerance traits (e.g., specific HSP alleles, antioxidant capacity, and TRP channel variants) for use in marker-assisted selection programs; and (3) conducting systems-level studies to quantify how integrated practices (e.g., rice–crayfish co-culture, specific probiotic regimens) affect the host’s transcriptomic, metabolomic, and microbiome profiles under thermal stress. Addressing these areas will bridge the gap between molecular mechanisms and farming practices, facilitating the design of next-generation, climate-resilient aquaculture systems for *Procambarus clarkii*.

## Figures and Tables

**Table 1:** Temperature ranges for the growth of various crayfish species.

Crayfish Species	Minimum Temperature (°C)	Optimal Temperature Range (°C)	Maximum Temperature (°C)	References
Red Swamp Crayfish (*Procambarus clarkii*)	16	20–25	32	[3]
European Crayfish (*Astacus astacus*)	10	18–22	25	[48]
Virile Crayfish (*Orconectes virilis*)	12	18–24	28	[49]
Robust Crayfish (*Cambarus robustus*)	10	16–22	24	[50]
Yabby (*Cherax destructor*)	10	18–24	30	[12]
Yellow Crayfish (*Faxonius luteus*)	13	17–22	25	[51]
Mississippi Crawfish (*Procambarus zonangulus*)	12	20–25	30	[51]
Redclaw (*Cherax quadricarinatus*)	20	24–30	34	[11,52]
Marbled crayfish or Marmorkrebs (*Procambarus virginalis*)	16	20–25	30	[53]
Rusty crayfish (*Orconectes rusticus*)	0.9–6.1	18–25	30.2–36.2	[51]
Porcelain crayfish (*Procambarus horsti*)	6.6 ± 0.7		28.6 ± 1.6	[51]
Virile crayfish (*Orconectes virilis*)	10	21	---	[39]
Spiny-cheek crayfish (*Procambarus spiculifer*)	8.1	23.4	33.3	[54]
Smooth marron (*Cherax tenuimanus*)	11	24	30	[51]

**Table 2:** Protein Names, Accession Numbers, and Functions of TRPA in Different Organisms.

Protein Name	Species	Function	References
ceTRPA1	*Caenorhabditis* *elegans*	Mechanosensation Cold sensation	[79] [85,86]
dTRPA1-A	*Drosophila* *melanogaster*	Heat and chemical sensation	[86,87]
dTRPA2-B	*Drosophila* *melanogaster*	Heat sensation	[87,88]
dTRPA3-C	*Drosophila* *melanogaster*	UV-nociception and the detector of electrophiles	[81,84,88,89]
dTRPA4-D	*Drosophila* *melanogaster*	Noxious thermosensor	[84]
AgTRPA1	*Anopheles* *gambiae*	Noxious thermosensor	[81]
AcTRPA1	*Anolis* *corolinensis*	Heat and noxious chemical sensation	[72,89]
XrTRPA1	*Xenopus* *ropicalis*	Heat and noxious chemical sensation	[72]

**Table 3:** Evidence Connecting TRP Channels to Temperature Sensing in Crayfish and Related Crustaceans.

Evidence Type	Species /Tissue	Key Finding	Implication	References
RNAi + infection assay at 32 °C	*Procambarus clarkii* (systemic)	Silencing PcTRPA1-1 impairs survival during bacterial challenge only at high temperature; TRPA1 expression is heat-induced.	TRPA1 functions as a heat sensor tied to thermal stress/immune responses.	[124]
Behavior (antenna hot-probe)	*Procambarus clarkii*	Rapid withdrawal to high heat; no robust response to cold or to capsaicin/isothiocyanate.	Specialized heat nociception in the antennal pathway.	[122]
Antennal neurophysiology	*Procambarus clarkii*	Transient bursts in antennal afferents to brief heat pulses.	Peripheral thermal transduction consistent with TRP gating.	[125]
Comparative genomics	Multiple crustaceans (including crayfish)	TRP families are broadly present; the temperature function is best supported for TRPA1 so far.	TRPA1 is the prime crayfish candidate; roles for TRPV/M remain open.	[80]
Cross-taxon TRPA1 reviews	Invertebrates & vertebrates	TRPA1 is polymodal and is frequently heat-sensitive in non-mammalian species.	Mechanistic template for crayfish TRPA1 heat gating.	[126,127,128,129]

**Table 4:** Integrated Molecular and Physiological Responses to Thermal Stress in *Procambarus clarkii*.

Stress Signal	Primary Sensor/Pathway	Key Molecular Effectors	Physiological/Biological Outcome	Farming Implication	References
Acute Heat	TRPA1 activation, Ca^2+^ influx	HSP70/90, Antioxidant enzymes	Protein stabilization, reduced oxidative damage, and modulated immune function	Selection for TRPA1/HSP alleles; probiotic use to support immunity	[69,108,122,133,134,135]
Chronic Cold	(Putative TRPM8/other sensors), Metabolic shift	Lipid metabolism genes, HSP20, Cold-shock proteins	Membrane fluidity maintenance, energy repartitioning, growth suppression	RAS temperature control; nutritional modulation of lipids	[69,107,129]
Combined Stress (Heat + Pathogen)	TRPA1-mediated immune sensing	Pro-phenoloxidase, antimicrobial peptides	Enhanced pathogen clearance at high temperature	Managing stocking density during heatwaves to reduce disease risk	[69,107,129]

**Table 5:** Crustacean species that are cultured in a Recirculation aquaculture system (RAS).

Species	Producer State	Water Used	References
*Homarus gammarus*	Norway	Salt water	[147]
	England		[148,151]
Crayfish, *Astacus astacus*	Germany	Freshwater	[151,152]
*Litopenaeus vannamei*	Indonesia	Salt water	[149]
	Germany		[150,153,154]
	America		[153,155,156,157,158]
	China		[23]
*Penaeus semisulcatus*	Turkey	Salt water	[158]
*Ibacus novemdentatus*	Japan	Salt water	[155]
*Penaeus latisulcatus*	Australia	Salt water	[158]
*Limulus polyphemus*	America	Salt water	[156]
*Callinectes sapidus*	America	Salt water	[159]
*Scylla serrata*	Indonesia	Salt water	[160]
*Portunus pelagicus*	Malaysia	Salt water	[48]
	Indonesia		[48]

**Table 6:** Summary of Key Nutritional Interventions to Mitigate Thermal Stress in *Procambarus clarkii*.

Intervention	Mechanism of Action	Physiological Benefits	References
Probiotics (*Lactobacillus*, *Clostridium butyricum*)	Modulate gut microbiota; enhance antioxidant capacity; regulate histamine metabolism.	Improved immune function, enhanced cold tolerance, reduced oxidative stress.	[134,167,168,169,170]
Biofloc	Provides protein, bioactive compounds, and a beneficial microbial consortium.	Enhanced immune and antioxidant enzyme activity; improved gut health.	[171,172]
Dietary Lipids (Optimal fatty acid profile)	Maintain membrane fluidity and composition.	Supports cellular homeostasis during cold stress; improves growth and gonad maturation.	[116,171]
Amino Acid Supplementation (e.g., Valine, Isoleucine)	Provide substrates for HSP synthesis and energy metabolism (gluconeogenesis, TCA cycle).	Fuels metabolic adaptation and stress protein production during thermal stress.	[123,167,173,174]

## Data Availability

No new data were created or analyzed in this study. Data sharing is not applicable to this article.
